# Supplementation of Forskolin and Linoleic Acid During IVC Improved the Developmental and Vitrification Efficiency of Bovine Embryos

**DOI:** 10.3390/ijms26094151

**Published:** 2025-04-27

**Authors:** Peipei Zhang, Hang Zhang, Muhammad Shahzad, Hubdar Ali Kolachi, Yupeng Li, Hui Sheng, Xiaosheng Zhang, Pengcheng Wan, Xueming Zhao

**Affiliations:** 1 State Key Laboratory of Sheep Genetic Improvement and Healthy Breeding, Institute of Animal Husbandry and Veterinary Sciences, Xinjiang Academy of Agricultural and Reclamation Sciences, Shihezi 832061, China; zhangphappy@163.com; 2 Tianjin Key Laboratory of Animal Molecular Breeding and Biotechnology, Tianjin Engineering Research Center of Animal Healthy Farming, Institute of Animal Science and Veterinary, Tianjin Academy of Agricultural Sciences, Tianjin 300381, China; 15910772142@163.com (Y.L.); shengjhui@163.com (H.S.);; 3 Institute of Animal Sciences (IAS), Chinese Academy of Agricultural Sciences (CAAS), No. 2 Yuanmingyuan Western Road, Haidian District, Beijing 100193, China; 82101215397@caas.cn (H.Z.); drmshahzad@gmail.com (M.S.); h.alikolachi@gmail.com (H.A.K.)

**Keywords:** lipid, embryo, vitrification, bovine

## Abstract

The success of assisted reproductive technology is contingent upon the growth potential of embryos post-vitrification process. When compared to in vivo embryos, it has been found that the high intracellular lipid accumulation inside the in vitro-derived embryos results in poor survival during vitrification. Based on this finding, the present study assessed the impact of incorporating forskolin and linoleic acid (FL) entering in vitro culture (IVC) on the embryos’ cryo-survival, lipid content, and viability throughout vitrification. Lipid metabolomics and single-cell RNA sequencing (scRNA-seq) techniques were used to determine the underlying mechanism that the therapies were mimicking. It was observed that out of 726 identified lipids, 26 were expressed differentially between the control and FL groups, with 12 lipids upregulated and 14 lipids downregulated. These lipids were classified as Triacylglycerol (TG), Diacylglycerol (DG), Phosphatidylcholine (PC), and so on. A total of 1079 DEGs were detected between the FL and control groups, consisting of 644 upregulated genes and 435 downregulated genes. These DEGs were significantly enhanced in the arachidonic acid metabolism, lipolysis, fatty acid metabolism, cAMP signaling pathway, and other critical developmental pathways. Based on the observation, it was concluded that forskolin and linoleic acid decreased the droplet content of embryos by modulating lipid metabolism, thus enhancing the vitrified bovine embryos’ cryo-survival.

## 1. Introduction

The cryopreservation of embryos is an effective method to extend the accessibility of valuable germplasm resources, streamline the process of long-distance transportation [1], and mitigate the potential risk associated with venereal disease transmission to a certain degree [2]. This technique allows for storing surplus oocytes and embryos for later use [3], leading to increased pregnancies from a single stimulation and decreased breeding costs and animal manipulation [4]. Gene banks have extensively adopted this technique to protect the native breeds of animal species from extinction and the potential threat posed by disease outbreaks [5,6].

The abundance of lipid droplets in livestock embryos is influenced by the culture environment; thereby, in vitro embryos have higher levels compared to in vivo embryos [7,8]. Moreover, excessive lipid droplet accumulation in embryos intensifies lipid peroxidation during cryopreservation, resulting in oxidative stress, apoptosis, lower blastocyst rate, and compromised cryo-tolerance [9,10]. Therefore, consistent efforts have been made to lower the amount of lipid droplets in developing embryos to improve their cryo-tolerance.

Adenylate cyclase, an enzyme that triggers endogenous lipase via 3’,5’-cyclic adenosine monophosphate (cyclic adenosine monophosphate, cAMP), is activated in cells by forskolin, a diterpene, allowing the release of fatty acids and glycerol [11,12]. It has been indicated that forskolin can induce lipolysis in oocytes and embryos, and improve their survival rate after vitrification [9,13,14]. Studies have demonstrated that forskolin significantly improved the developmental competence of porcine [15], bovine [16], horse [17], and sheep [18] oocytes. In addition, it was shown that when 10 μM forskolin was added to the in vitro culture (IVC) medium, it caused a higher rate of blastocyst development [19]. Meanwhile, the supplementation of forskolin during early embryonic porcine and cattle development in porcine and cattle was enough to reduce the lipid content in blastocysts, thereby improving their low-temperature tolerance [14,20].

Linoleic acid is a long-chain polyunsaturated fatty acid that reduces free fatty acid uptake without increasing lipolysis [21]. Linoleic acid has been found to pass through the lipid bilayer of the plasma membrane in preimplantation embryonic cells and to enter lipid droplets during IVC [22], thereby enhancing the fluidity of embryonic cell membranes and improving the tolerance of embryos to cryopreservation [23]. It has been shown that the addition of linoleic acid alters the intracellular fatty acid content and improves the developmental competence of bovine [22,24] and ovine [25] embryos after vitrification [26].

Consequently, the quality and survival rate of embryos following vitrification may be improved by adding metabolic regulators to the culture medium. This study aimed to investigate the effects of forskolin and linoleic acid on apoptosis, lipid content, ROS levels, and cytoskeletal structure in vitrified bovine oocytes. Through the utilization of lipidomic and transcriptomic techniques, we sought to elucidate the mechanisms by which forskolin and linoleic acid impact lipid metabolism in embryos. Therefore, the study aimed to conceive a strategy to decrease abnormal lipid accumulation in bovine embryos to maintain their development potential following vitrification.

## 2. Results

### 2.1. Effect of Forskolin on the Developmental Ability and Survival Rate of Bovine Embryo

Table 1 showed that the rates of cleavage blastocyst formation of the 10 µM forskolin group (91.67 ± 3.71%, 42.42 ± 4.85%) were greater than those of the control group (81.90 ± 2.57%, 36.89 ± 4.47%), 5 µM forskolin group (84.87 ± 4.65%, 35.64 ± 4.70%), and 15 µM forskolin group (64.18 ± 4.32%, 27.91 ± 3.81%). Meanwhile, the survival rate of the 10 µM forskolin group (95.24 ± 7.23%) was greater than the control group (86.67 ± 6.59%), the 5 µM forskolin group (88.89 ± 7.35%), and the 15 µM forskolin group (83.33 ± 7.06%).

### 2.2. Effect of Linoleic Acid on the Developmental Ability and Survival Rate of Bovine Embryo

Table 2 showed that the cleavage and blastocyst rates of the 100 µM linoleic acid group (88.74 ± 6.73%, 38.81 ± 2.55%) were greater than those of the control group (80.00 ± 6.59%, 33.33 ± 2.41%), 50 µM linoleic acid group (61.17 ± 5.85%, 27.83 ± 1.68%), 500 µM linoleic acid group (60.54 ± 5.68%, 29.21 ± 1.84%). Meanwhile, the survival rate of the 100 µM linoleic acid group (96.15 ± 8.53%) was greater than those of the control group (86.36 ± 7.15%), 50 µM linoleic acid group (81.25 ± 7.46%) and 500 µM linoleic acid group (80.77 ± 7.21%, *p* < 0.05).

### 2.3. Effect of Forskolin and Linoleic Acid on the Developmental Ability and Survival Rate of Bovine Embryo

Table 3 revealed the F-L group’s cleavage rate and blastocyst rate (89.54 ± 7.62%, 45.26 ± 5.24%) were greater than those of the control group (75.57 ± 7.46%, 30.30 ± 2.65%), forskolin group (82.39 ± 8.52%, 39.74 ± 4.12%) and linoleic acid group (83.08 ± 8.46%, 37.97 ± 4.03%). Meanwhile, compared to the control group (80.65 ± 7.68%), the forskolin group (90.24 ± 7.31%), and the linoleic acid group (89.47 ± 8.15%, *p* < 0.05), the F-L group had a greater survival rate (98.18 ± 8.64%, *p* < 0.05).

### 2.4. Effect of Forskolin and Linoleic Acid on Lipid Droplet Content in the Bovine Embryo

**Figure 1:**
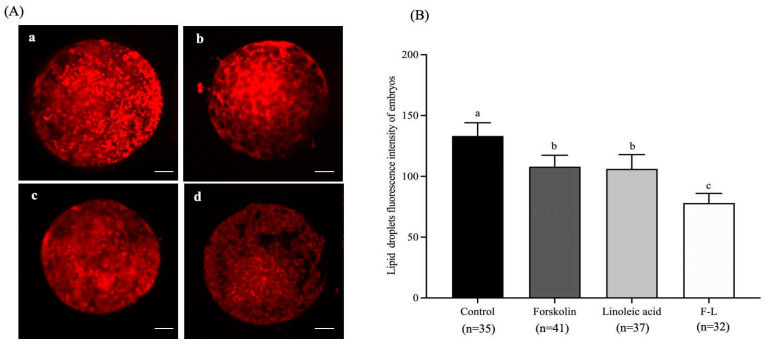
Effect of forskolin and linoleic acid on the lipid droplet content of bovine embryos. (**A**) Fluorogram of lipid droplet staining of embryos; a: control group, b: forskolin group, c: linoleic acid group, d: F-L group. (**B**) Effect of forskolin and linoleic acid on the amount of lipid droplet content of bovine embryos. Scale bar = 50 μM. Values with different superscripts indicate differences between groups (*p* < 0.05).

As illustrated in Figure 1, the fluorescence intensity of the lipid droplets in the F-L group (78.14 ± 6.98) was significantly lower than that of the control group (133.22 ± 9.75), forskolin group (108.05 ± 8.35), and linoleic acid group (106.18 ± 6.98, *p* < 0.05).

### 2.5. Metabolomic Analysis of Lipid Metabolites in Bovine Embryos Combined Treatment with Forskolin and Linoleic Acid

**Figure 2:**
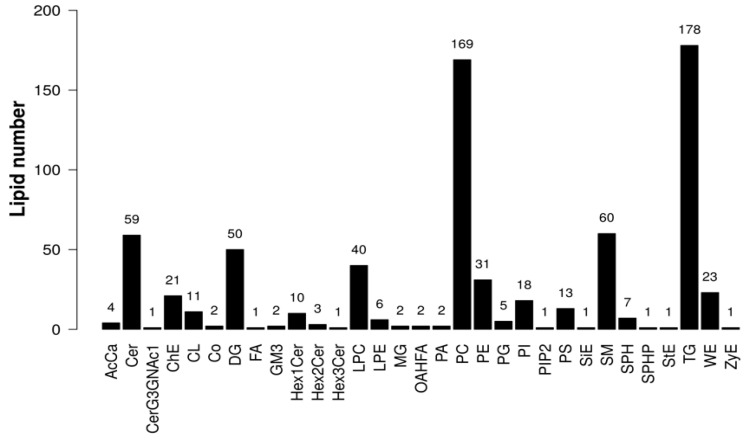
Statistical analysis of lipid class and lipid species.

**Figure 3:**
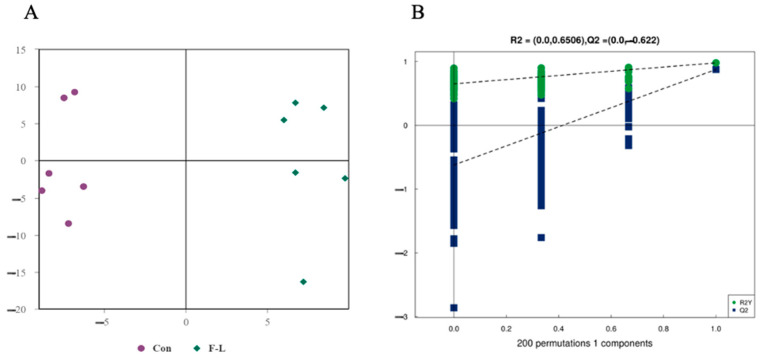
Score scatter plot and permutation test of the OPLS-DA model. (**A**) Score plots were derived from the LC-MS/MS data of F-L and the control lipidomic. The x-axis and y-axis represent the first and second principal components, respectively. The purple and green colors indicate biological duplication within their respective groups. (**B**) The permutation tests based on LC-MS/MS data. In these tests, the x-axis and y-axis represent the correlation coefficient and value of R^2^Y and Q^2^, respectively.

The results of LC-MS/MS analysis showed 31 classes of lipids and 726 lipids, and the proportions of these lipids are shown in Figure 2. The main lipids were Triacylglycerol (TG), Phosphatidylcholine (PC), Sphingomyelin (SM), Ceramide (Cer), and Diacylglycerol (DG). The multivariate statistical analysis of lipid alterations between the F-L and control groups was performed using OPLS-DA. As shown in Figure 3, the samples from the two groups were completely separated along the *x*-axis, and the model parameters were R^2^Y = 0.6506 and Q^2^ = 0.622, indicating that the model was robust and not overfitted. The stability of the model was verified using a permutation test, and the slope of the regression line for the permutation test was positive, demonstrating the excellent degree of fit and strong predictive power of the OPLS-DA model.

### 2.6. Differential Lipids Between F-L and Control Groups

To further investigate linoleic acid and forskolin effects on embryonic lipid metabolism, the two groups were screened for differential lipid molecules. 

**Figure 4:**
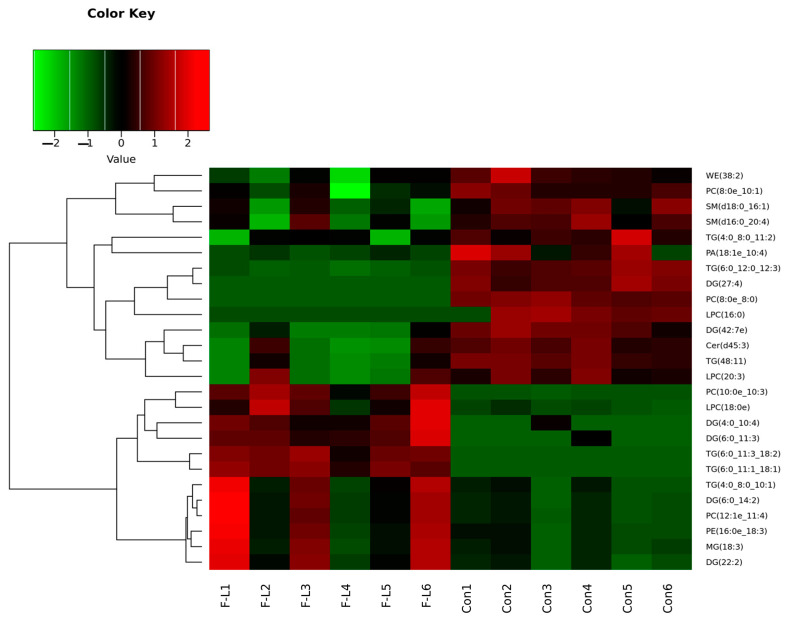
Clustering heatmap of significantly different lipid molecules. Note: Each column represents a group of samples, and each row represents a differential lipid molecule.

A total of 26 lipid molecules (VIP > 1 and *p* < 0.05) were found to be significantly unlike between the F-L group and the control group (Table 4). Then, the clustering analysis results of the differential lipids are shown in Figure 4. These included 12 lipids that were seen significantly upregulated and 14 lipids that were seen significantly downregulated in the F-L group. These lipid molecules were mainly attributed to TG, DG, Phosphatidic acid (PA), Lysophosphatidylcholine (LPC), PC, and Phosphatidylethanolamine (PE).

### 2.7. Differentially Expressed Genes (DEGs) in Forskolin and Linoleic Acid Treated Bovine Embryos

**Figure 5:**
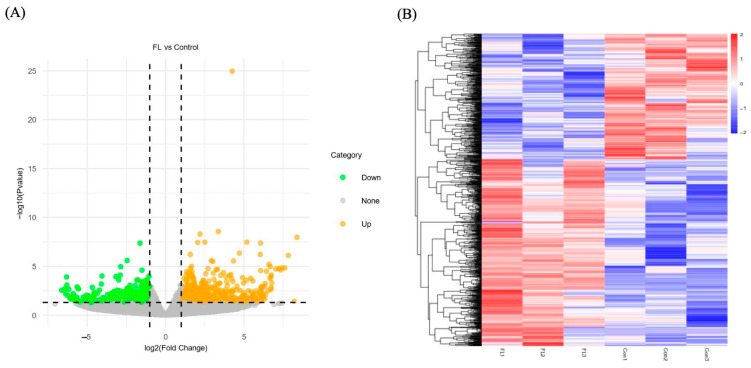
Screening of differential expressions of mRNA. (**A**) Volcano plot of DEGs in FL and Con groups. (**B**) Clustering analysis of DEGs. Red indicates high expression, and blue indicates low expression.

The volcano plots were utilized to clarify the differences in gene expression levels between the FL and control groups (Figure 5A). Subsequently, a clustering analysis was conducted on DEGs based on their expression patterns, and the findings indicated that the samples in the same group were well clustered together (Figure 5B). Comparing the FL group to the control group, 644 genes were upregulated and 435 were downregulated. Some of those DEGs were listed in Table 5, including microtubule-related (*TUBA4A*), apoptosis genes (*CASP7*), oxidative stress genes (*CAT*, *GPX7*), embryo development (*SEC22B*, *NDRG4*, *MX2*), lipid metabolism-related (*ACSL1*, *DECR1*, *ACADL*, *CDKN2C*, *PPARA*).

### 2.8. GO Enrichment Analysis of the DEGs

**Figure 6:**
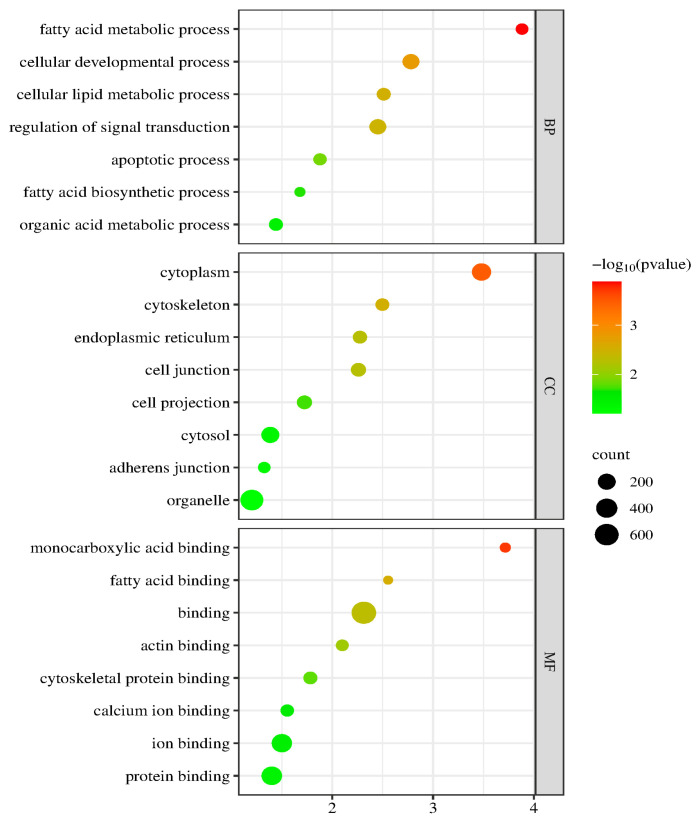
GO enrichment analysis of the DEGs. The spot color represents the FDR, and the spot size represents the enriched gene number.

Figure 6 shows the GO terms with a significant enrichment of DEGs between the FL and control groups. The GO enrichment analysis revealed that the biological process (BP) mainly consisted of fatty acid metabolic, cellular lipid metabolic, and fatty acid biosynthetic processes. Cell components (CCs) primarily involve the cytoplasm, endoplasmic reticulum, cytoskeleton, and organelles. Molecular function (MF) was primarily associated with monocarboxylic acid binding, fatty acid binding, cytoskeletal protein binding, and protein binding.

### 2.9. Pathway Enrichment Analysis of DEGs

**Figure 7:**
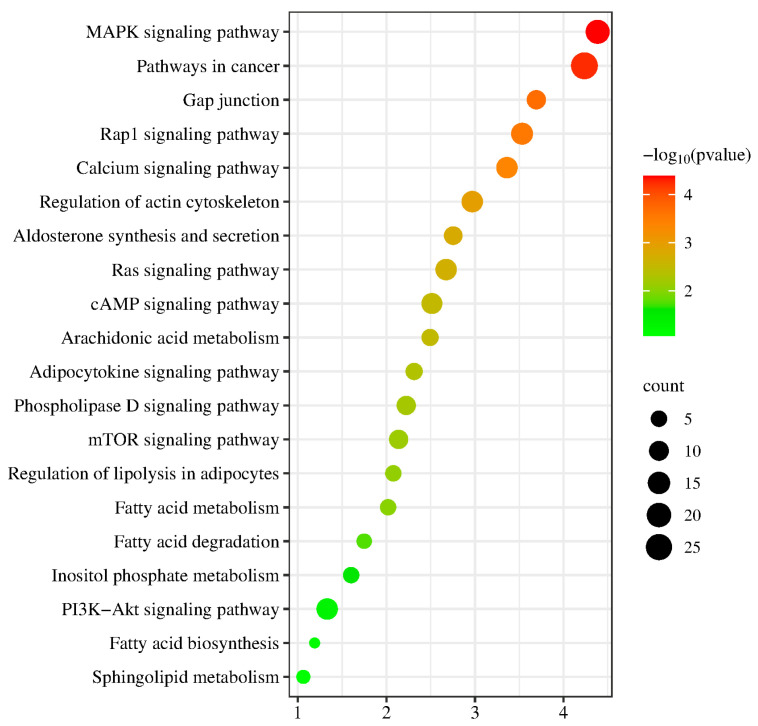
KEGG pathway analysis of the DEGs. Results from KEGG analysis; the color of each dot indicates the *p* value of the two sets of DEGs, and the size of the dot indicates the number of DEGs in each KEGG pathway.

As revealed in Figure 7, DEGs associated genes between the FL and Con groups were mostly enhanced with the MAPK, calcium, and cAMP signaling pathways, as well as the breakdown and metabolism of fatty acids, Inositol phosphate metabolism, and arachidonic acid metabolism.

### 2.10. Effect of Forskolin and Linoleic Acid on Gene Expression in Bovine Blastocysts

**Figure 8:**
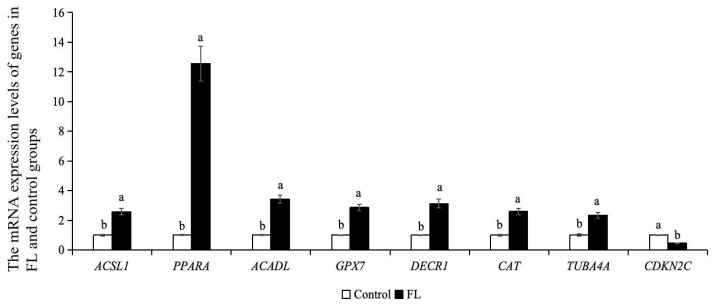
Effect of forskolin and linoleic acid on gene expression in bovine blastocysts. a, b: Values with different superscripts represent significant differences between groups (*p* < 0.05).

As shown in Figure 8, the mRNA expression levels of lipid metabolism genes (*ACSL1*, *DECR1*, *ACADL*, *PPARA*), microtubule genes (*TUBA4A*), and oxidative stress genes (*CAT*, GPX7) in the blastocysts of the FL group were significantly higher than control group. The mRNA expression level of the *CDKN2C* in the FL group was lower than that of the control group (*p* < 0.05).

### 2.11. Effect of Forskolin and Linoleic Acid on the Apoptosis of Bovine Blastocysts

**Figure 9:**
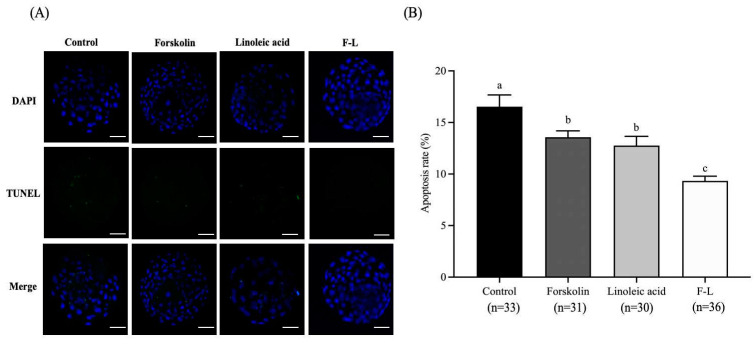
Forskolin and linoleic acid on the apoptosis of bovine embryos. Note: (**A**) Fluorogram of TUNEL-stained embryos. (**B**) Forskolin and linoleic acid on the apoptosis of bovine embryos. Scale bar = 50 μM. Values with different superscripts indicate differences between groups (*p* < 0.05).

As shown in Figure 9, the apoptosis rate of blastocysts of the F-L group (9.34 ± 0.41%) was significantly lower than the control group (16.53 ± 1.02%), forskolin group (13.58 ± 0.55%), and linoleic acid group (12.76 ± 0.80%; *p* < 0.05).

### 2.12. Linoleic Acid and Forskolin’s Effects on the ROS Levels of Bovine Blastocysts

**Figure 10:**
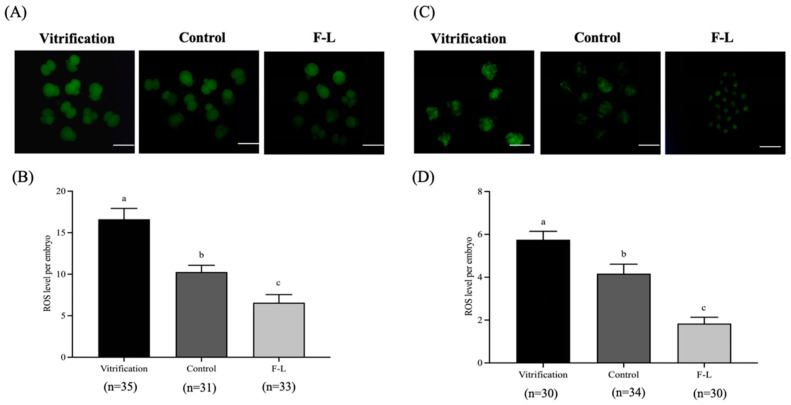
Forskolin and linoleic acid on ROS levels in bovine embryos. (**A**) Representative images of bovine 2-cell ROS staining. (**B**) Forskolin and linoleic acid on the ROS levels of bovine 2-cell embryos. (**C**) Representative images of bovine embryo ROS staining; (**D**) forskolin and linoleic acid on the ROS levels of bovine embryos. Scale bar = 50 μM. Values with different superscripts indicate differences between groups (*p* < 0.05).

As revealed in Figure 10, the ROS levels of 2-cell and blastocysts of the F-L group (6.58 ± 0.87; 1.84 ± 0.26) were significantly lower than the control (10.27 ± 0.72; 4.17 ± 0.39) and vitrified group (16.62 ± 1.18; 5.75 ± 0.35, *p* < 0.05).

### 2.13. Effect of Forskolin and Linoleic Acid on the Cytoskeleton of Bovine Blastocyst

**Figure 11:**
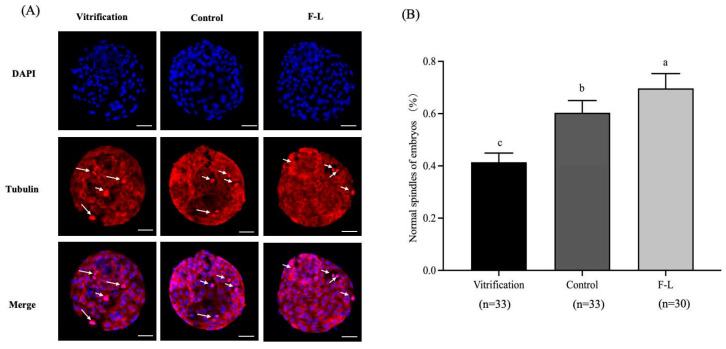
Forskolin and linoleic acid on the cytoskeleton of bovine embryos. (**A**) Fluorogram of Tubulin-stained embryos; short arrows: normal spindle chromosome configurations; long arrows: abnormally shaped spindles; DAPI staining showed cell nuclear (blue color) and Tubulin staining showed microtube (red color). (**B**) Cytoskeletal analysis in vitrification and control and F-L group bovine embryos. Scale bar = 50 μM. Values with different superscripts indicate differences between groups (*p* < 0.05).

The normal spindle rate of blastocysts in the F-L group (69.68 ± 5.06%), as shown in Figure 11, was substantially greater than that of the vitrified group (41.44 ± 3.12%) and control group (60.32 ± 4.21%, *p* < 0.05).

## 3. Discussion

Forskolin is recognized for its lipolytic properties and is commonly utilized as a lipid modulator [27]. It elevates intracellular cAMP levels by stimulating adenylate cyclase (AC) [9], subsequently activating cAMP-dependent protein kinase A (PKA), which phosphorylates intracellular lipase and enhances fatty acid β-oxidation [28]. Forskolin supplementation has demonstrated the ability to improve cryotolerance in oocytes and embryos of various species [29]. The current investigation’s findings showed that the administration of forskolin notably enhanced the rates of cleavage, blastocyst formation, and viability after vitrification cryopreservation in bovine embryos. This observation aligns with similar trends observed in porcine oocytes [30] and bovine embryos [31].

Linoleic acid, a crucial polyunsaturated fatty acid, plays an important part in the embryo’s development and the maturation of mammalian oocytes [32]. Previous research indicates that adding linoleic acid to the medium modifies the lipid composition of oocytes for IVM and enhances the development of sheep [33], porcine [34], and bovine embryos [35]. Coherent with previous reports, the results of the present study showed that linoleic acid treatment increased the cleavage, blastocyst rate, and cryo-tolerance of bovine embryos. Moreover, it was also revealed that the supplementation of forskolin and linoleic acid to IVC could synergistically improve the development of blastocysts and their cryotolerance in bovine embryos.

Forskolin can activate AC to increase intracellular cAMP levels, inducing the activation of intracellular lipase, thus promoting lipolysis with the release of glycerol and fatty acids [20]. Similarly, we found that the forskolin addition to the IVC medium significantly reduced the intracellular lipid content in bovine embryos. Conjugated linoleic acid enhances the fluidity of linoleic acid and facilitates the synthesis of TG and phospholipid, thus improving the lipid profile of adipocytes [36]. Therefore, the supplementation of forskolin and linoleic acid to the IVC medium significantly reduces intracellular lipid accumulation by improving the overall lipid metabolism of developing embryos.

TGs are a class of lipids in mammalian cell cytoplasm stored as droplets, providing energy for early embryonic development [37]. During oocyte maturation, growth factors, hormones, and serum are added, which results in oxidative metabolism, oxidizing fatty acids from TG breakdown in the tricarboxylic acid cycle [38,39]. Therefore, IVC conditions can potentially elicit an abundance of oxidized glycerophospholipids. So, changes in lipid profiles of embryos may be linked to IVC conditions. DG represents a category of structural lipids characterized by the replacement of a fatty acid with a hydroxyl group in TG. It is a crucial intracellular second messenger that plays a role in various cellular reactions. They also promote fatty acid oxidation processes, modulating lipid metabolism and regulating gene expression linked with lipid metabolism [40]. The most prevalent phospholipid in mammalian cells is PC, having a vital role in cell structure and cellular activities, including vesicular transport, signal transduction, and regulation of lipoprotein secretion [41]. Forskolin activates intracellular lipases for lipolysis [20], and linoleic acid enters cells to synthesize triacylglycerols and phospholipids, which enhance the lipid profile of lipocytes [36]. Membrane proteins control the uptake of lipid metabolites and fatty acids from the culture medium. However, release occurs predominantly via passive diffusion [42]. In this case, as shown in our results, the TG and DG content in the F-L group is greater than the control one, and the products of lipolysis can be partially released into the embryonic culture medium to regulate lipid metabolism.

In this study, we compared transcriptomics of the FL and control group embryos using scRNA-seq technology. Several genes related to lipid metabolism (*ACSL1*, *DECR1*, *ACADL*) were noted to be upregulated in the FL group compared to the control group. Fatty acid elongation, oxidative catabolism, phospholipogenesis, and protein acylation are all aspects of lipid metabolism that are regulated by the important enzyme *ACSL* [43] and oversee fatty acid acylation and activation upon cell entry [44]. It has been evident that upregulation of the *ACSL1* gene reduces lipid peroxidation and decreases ROS through lipid reprogramming [45]. Further, KEGG analysis revealed that the gene *ACSL1* is enriched with fatty acid degradation and fatty acid metabolism pathways. *DECR1* is a mitochondrial enzyme that regulates the balance between saturated phospholipids and unsaturated ones in the auxiliary pathway of β-oxidation, which maintains redox homeostasis in vivo, and the absence of *DECR1* impairs lipid metabolism [46]. *ACADL* is a mitochondrial enzyme that catalyzes fatty acid oxidation [47]. It was found that the gene *ACADL* is enriched in the fatty acid degradation pathway by KEGG analysis, which explains the reduction in lipid content in the FL group.

The oxidative stress-related gene *GPX7* was observed to be significantly upregulated in the FL group. *GPX* family member *GPX7* is an antioxidant enzyme that reduces ROS production and limits their toxicity [48]. *GPX7* is a marker for endoplasmic reticulum and oxidative stress, and any change in *GPX7* activity indicates how the body reacts to oxidative stress [49]. It is established that *GPX7* can ameliorate nonalcoholic steatohepatitis by regulating oxidative stress levels [50]. *GPX7* participates in the arachidonic acid metabolic pathway. Through current research work, it was found that *GPX7* is enriched in this pathway according to KEGG analysis. An omega-6 polyunsaturated fatty acid named arachidonic acid is a part of the signaling cascade that determines a cell’s destiny in fetal placental development and has an enhanced role in the immunomodulatory effects of angiogenesis in embryonic stem cells and mesenchymal stem cells (MSCs) [51]. Research findings indicate that arachidonic acid plays a significant role in embryonic development, overall health, brain development, and immune response [52]. *CDKN2C*, a gene associated with lipid metabolism, was shown to be downregulated in the FL group compared to the control group. *CDKN2C* has been associated with inhibition of the cell cycle during adipocyte differentiation [53] and can prevent G1 by inhibiting cyclin-dependent kinase 4 or 6 (CDK4/6) onset. *CDKN2C* deficiency decreases lipid stock in adipocytes during the differentiation process and decreases gene expression, which regulates adipocyte function [54].

AC catalyzes the extraction of pyrophosphate from ATP, producing cAMP [55]. It can be deactivated by hydrolysis by phosphodiesterase (PDE) [56]. AC and PDE interact to regulate intracellular cAMP levels and activate β-oxidative regulation in mitochondria [57]. It has been reported that forskolin, as an activator of the cAMP pathway, can increase cAMP levels and maintain oocyte meiotic arrest in sheep [58], bovine [59], and mice [60] oocytes. Moreover, *PPAR* has been shown to mediate the stimulatory PGI2 effects on embryonic development and blastocyst hatching, as well as to contribute to embryonic cell proliferation [61]. Our results showed that *PPARA* was upregulated in the FL group, which is intricate in the β-oxidation of fatty acids regulation. Therefore, enhancing lipid metabolism in embryos contributes to the establishment of an optimal cellular environment for embryonic development [62]. At the same time, *PPARA* is also enriched in the signaling pathway cAMP, recommending that forskolin and linoleic acid may increase the cAMP level by regulating the expression of *PPARA* and thus reduce the intracellular lipid content.

Apoptosis is a key parameter in embryo quality assessment and has a key role in embryonic development. It has been observed that the rate of apoptosis remains higher in vitrified embryos in contrast to fresh embryos [63,64]. Reducing lipid content before cryopreservation could potentially aid in preserving embryo cytoplasm, boosting lipid metabolism gene expression, and reducing apoptosis in embryos [20,23]. Research indicates that forskolin reduces lipid deposition in porcine and bovine embryos, promoting cryotolerance and diminishing apoptosis [13,20]. It has been shown that linoleic acid modulates NF-κB, which reduces apoptosis in porcine embryos [34], and improves embryo development [65]. Our current experiments demonstrated a significantly reduced rate of apoptosis in the F-L group, compared to forskolin, linoleic acid, and the control. Collectively, these results provide indications that forskolin and linoleic acid act cooperatively to reduce lipid deposition in bovine embryos during the preimplantation period, thereby decreasing apoptosis.

Vitrification induces lipid peroxidation in embryos, leading to the impairment of essential biomolecules, including DNA, proteins, and plasma membrane [66]. It also disrupts the redox state balance, leading to a hike in ROS and poor embryo survival [67]. Forskolin exhibits antioxidant properties and decreases *Bax* and *Caspase-3* expression, thereby significantly lowering cellular ROS levels [68]. Similarly to Cai [47], who demonstrated forskolin efficacy in reducing ROS in vitrified embryos, our findings indicate that forskolin and linoleic acid significantly lower ROS in bovine embryos. This reduction is linked to the upregulation of antioxidant-related genes (*CAT* and *GPX7*) (Table 5), which encode critical enzymes in the antioxidant defense mechanism, safeguarding cells from oxidative stress-induced damage.

Vitrification damages the embryo cytoskeleton [69] by microtubule depolymerization [70]. Our transcriptomic analysis revealed upregulation of the microtubule-related gene (*TUBA4A*) in the FL group, with GO enrichment analysis indicating that differential genes were enriched in the cytoskeleton. Our results suggest that forskolin and linoleic acid enhance embryo spindle morphology and quality. Previous findings have shown that forskolin elevates intracellular cAMP levels, which activate PKA, resulting in Rho inactivation, which aids in preserving cytoskeletal integrity and normal cellular morphology [71].

## 4. Materials and Methods

Unless otherwise specified, all chemicals used in this study were purchased from Sigma-Aldrich (St. Louis, MO, USA), and plasticware was obtained from Corning Inc. (Corning, NY, USA).

### 4.1. Oocytes In Vitro Maturation (IVM)

From the slaughterhouse, bovine ovaries were collected and dispatched to the laboratory within 2 h at 37 °C. Cumulus oocyte complexes (COCs) were aspirated from follicles with a diameter of 2–8 mm, and only those with uniform cytoplasm and at least three complete layers of tightly packed cumulus cells were chosen for IVM. 10 μg/mL estradiol, 10% (*v*/*v*) fetal bovine serum (FBS; Gibco BRL Division), 10 μg/mL follicle-stimulating hormone (FSH), 10 μg/mL heparin (HP), and 10 μg/mL luteinizing hormone (LH) were added to a HEPES-buffered M199 (Gibco BRL; Grand Island, NY, USA) that served as the IVM solution. For 22–24 h, the COCs were cultivated at a temperature of 38.5 °C with 5% CO_2_ and high humidity.

### 4.2. In Vitro Fertilization (IVF)

After oocyte culture, IVF was performed as previously reported by Brackett and Oliphant [72]. Briefly, for 30 s, the frozen sperm underwent thawing at 37 °C. The post-thaw semen was then rinsed twice at 600× *g* for 5 min in the Brackett and Oliphant (BO) medium, which contained penicillin, heparin, streptomycin, and bovine serum albumin (BSA). A total of 1 × 10^6^ spermatozoa per milliliter was the final concentration following resuspension. A total of 20–30 oocytes were added to each drop of fertilization media, and the gametes were incubated for 8–18 h at 38.5 °C in humidified air containing 5% CO_2_.

### 4.3. IVC

After 16–18 h, the surrounding cumulus cells and sperm were taken away from the zygotes through a mouth pipette. After that, forskolin and linoleic acid were added as follows: (1) control group: CR1aa basal culture medium; (2) forskolin group: 10 μM forskolin addition for 24 h in CR1aa medium; (3) linoleic acid group: 100 μM add linoleic acid was added to CR1aa medium; (4) F-L (or FL) group: 10 μM forskolin (24 h) and 100 μM linoleic acid group were added to CR1aa medium. The embryos were cultured at 38.5 °C and 5% CO_2_ until day 7 after the initiation of fertilization.

### 4.4. Vitrification and Warming

The thawing and vitrification procedures were slightly modified from Zhao et al. [73]. Briefly, the embryos were initially immersed in vitrification solution with 10% ethylene glycol (EG) and 10% Dimethyl sulfoxide (DMSO) for 30 s. Then, embryos were transferred to EDFSF40 (20% DMSO, 20% EG, 300 g/L Ficoll, 20% FBS, and sucrose concentration 0.5 M) for 25 s. The embryos were immediately plunged into liquid nitrogen after being loaded into open-pulled straw (OPS).

The OPS tubes were taken out from liquid nitrogen for the warming process, and the embryos in the tubes were blown into sucrose solution 0.25 M and incubated for about 5 min. Following that they were shifted to a sucrose solution of 0.25 M and incubated for about 5 min. Afterward, the embryos were cultured at 38.5 °C under 5% CO_2_.

### 4.5. Embryo Lipid Droplet Staining

With minor adjustments according to the previously described method [74], embryos were rinsed three times with PBS containing 0.1% polyvinyl alcohol (PVA), fixed with 4% paraformaldehyde fixative for 30 min at room temperature. Following three rounds of washing with 0.1% PVA/PBS, the embryos were incubated in a 10 μg/mL Nile Red staining solution at 37 °C for 2 h before being examined using an epifluorescence inverted microscope (Nikon, Tokyo, Japan). The fluorescent images were statistically analyzed utilizing ImageJ software version 1.8.0 (NIH, Bethesda, MD, USA).

### 4.6. Assessment of the Apoptotic Index

Following a protocol similar to Khan et al. [75], with slight adjustments, apoptosis was examined utilizing a TUNEL (terminal deoxynucleotidyl transferase biotin-dUTP nick end labeling) test kit (Solarbio, Beijing, China) in compliance with the guidelines provided by the manufacturer. Briefly, the blastocysts were rinsed with 0.1% PVA/PBS, then fixed at room temperature for 30 min using 4% paraformaldehyde, and permeabilized with 0.1% Triton X-100. Following three washes with 0.1% PVA/PBS, embryos were placed in an incubator for 1 h at 37 °C in the dark with TUNEL equilibration and the terminal deoxynucleotidyl transferase enzyme. After that, they received treatment for 5 min at 37 °C with 1 mg/mL DAPI. Following that, a confocal laser microscope (Leica, Wetzlar, Germany) was used to capture the images, and ImageJ software version 1.8.0 (NIH, Bethesda, MD, USA) was used to analyze the images for the intensity of fluorescence.

### 4.7. Embryo ROS Staining

With some modifications to the method described by García-Martínez et al. [76], intracellular ROS levels in embryos were quantified by labeling embryos that were washed twice in 0.1% PVA/PBS, then incubated in PVA/PBS supplemented with 5 μM 2’,7’-dichlorodihydrofluorescein diacetate (H2DCFDA) for 30 min at 38.5 °C in a humidified 5% CO_2_ air atmosphere. Then, embryos were observed on the epifluorescence inverted microscope (Nikon, Tokyo, Japan). ImageJ software version 1.8.0 (NIH, Bethesda, MD, USA) was utilized for fluorescence intensity.

### 4.8. Embryo Cytoskeleton Staining

According to a previous method described by Cai et al. [77], the embryos were fixed with 4% paraformaldehyde at room temperature for 30 min, then washed 3 times with 0.1% TritonX-100 for 5 min each time. The embryos were placed in Tubulin-Tracker Red solution (C1050, Beyotime, Shanghai, China) at room temperature and incubated for 30 min in the dark. After that, they were washed twice in 0.1% PVA/PBS and further incubated in 1 mg/mL DAPI at 37 °C for 5 min. Then, embryos were fixed on a glass slide for examination under the confocal laser microscope (Leica, Wetzlar, Germany). ImageJ software version 1.8.0 (NIH, Bethesda, MD, USA) was utilized for fluorescence intensity.

### 4.9. Untargeted Lipidomics Analysis

With slight modifications based on the previously described method [78], the embryo samples were first added, and 200 μL of water and 240 μL of methanol were used to homogenize them. After adding 800 μL of MTBE, the mixture was sonicated for 20 min at 4 °C, followed by allowing the mixture to settle for 30 min at room temperature. Then, the solution was centrifuged at 14,000× *g* for 15 min at 10 °C, supernatant was obtained from the upper layer of the organic phase and dried under nitrogen gas. For mass spectrometry analysis, a solution of 200 μL consisting of 90% isopropanol/acetonitrile was introduced and vigorously vortexed. Further, 90 μL of the solution for 15 min at 10 °C was centrifuged at 14,000× *g*, followed by collection of the supernatant for analysis.

The Vanquish UHPLC system (Thermo Fisher Scientific) coupled with the Q-Exactive Plus system (Thermofisher Scientific) was used for untargeted lipidomics analysis. Utilizing a CSH C18 column (1.7 μM, 2.1 mm × 100 mm, Waters Corporation, Wexford, Ireland), chromatographic separation was carried out using an LC separation. The auto-sampler temperature was 4 °C. Solvent A (60% acetonitrile, 40% water) had 0.1% formic acid and 0.1 mM ammonium formate, and solvent B (10% acetonitrile + 90% isopropanol) used 0.1% formic acid and 0.1 mM ammonium formate. The elution gradient (0–3.5 min, 40% B; 3.5–13 min, 40–70% B; 13–19 min, 75–99% B; 19–40 min, 40% B). The Q-Exactive Plus system, with the ability to function with both positive and negative modes, was used to gather mass spectrometry data. For every measurement, the ESI parameters were refined and displayed in this fashion: 300 °C was the source temperature, 350 °C was the capillary temperature, 3000 V was the ion spray voltage, 50% was the S-Lens RF Level, and 200–18,000 *m*/*z* was the mass range for scanning.

### 4.10. Sample Preparation, Library Construction, and RNA Sequencing

The zona pellucida of the embryo was digested using a strepase enzyme. Subsequently, each embryo was treated with a lysis solution. The single-cell RNA sequencing (scRNA-seq) library construction and sequencing process adhered to the protocol detailed by Gao et al. [79] with minor adjustments. Individual embryos were lysed to extract RNA, which was later reversely transcribed into first-strand cDNA using a six-nucleotide random primer. The complementary second strand was synthesized in the presence of dNTPs through a DNA polymerase I reaction. The resulting double-stranded cDNA was cleaned using AMPure P magnetic beads, followed by PCR amplification to yield the final cDNA product. The qualified cDNA library was sequenced by the Illumina HiSeq 2500 platform, and the paired-end reads of 150 bp were obtained by the PE 150 bp technique. The original sequences were filtered using Trimmomatic (v0.39) software, and adapter sequences, low-quality bases, and contaminated sequences were removed. Then, HISAT2 (v2.1.0) was used to compare the filtered sequence with the bovine reference genome sequence (ARS-UCD2.0 genome). Then, DEseq2 (v1.20.0) was used to analyze the differences in gene expression between groups. |log2Ratio| ≥ 1 and *p* < 0.05 were considered as significant differences.

### 4.11. Functional Enrichment Analysis

In this research, an enrichment analysis was conducted on differentially expressed genes using Gene Ontology (GO), which categorizes genes into 3 terms: biological process, cellular component, and molecular function. The Kyoto encyclopedia of genes and genomes gene ontology (KEGG) database was used to perform functional enrichment analysis of the screened differential genes in order to identify biological pathways with substantial enrichment. Significantly enriched KEGG pathways and GO items are indicated by *p* < 0.05.

### 4.12. Quantitative Real-Time Polymerase Chain Reaction (qRT-PCR) of Genes

According to the manufacturer’s protocol, the Single Cell-to-CT quantitative real-time PCR kit (Life Technologies, Carlsbad, CA, USA) was used to measure the gene expression levels of blastocysts. The qRT-PCR was run using an ABI 7500 SDS instrument (Applied Biosystems, Foster City, CA, USA). The reaction took place at 95 °C for 2 min, then 40 cycles for 10 s at 95 °C and 30 s at 60 °C. Using β-actin as the reference gene, the folding changes in gene expression were analyzed by the 2^−44CT^ method. The PCR primers used in the assessment are shown in Table 6.

### 4.13. Statistical Analysis

All experiments were conducted at least three times. All data were subjected to one-way ANOVA using Duncan’s test with SAS software version 9.2.0 (SAS Institute, Cary, NC, USA).

## 5. Conclusions

The cocktail of forskolin and linoleic acid in the IVC medium improved bovine embryo development and survival rate after vitrification, reduced the content of lipid droplets, apoptosis, and ROS levels in bovine embryos. Similarly, forskolin and linoleic acid could alter the lipid metabolism, increased *PPARA* gene expression, and regulate the pathway cAMP signaling to promote β-oxidation of fatty acids, which altered the lipid metabolism process of embryos, decreased the lipid content of embryos to further enhance the freeze tolerance of embryos, and contributed to the enhancement developmental capacity of bovine embryos, and it contributes to the advancement of both the freezing survival rate and developing capacity of bovine embryos.

## Figures and Tables

**Table 1:** Effect of forskolin on the developmental ability and survival rate of bovine embryos.

Groups	No. COCs	Cleavage Rate	Blastocyst Rate	Survival Rate
0 μM	105	81.90 ± 2.57% (86/105) ^b^	34.89 ± 4.47% (30/86) ^b^	86.67 ± 6.59% (26/30) ^b^
5 μM	119	84.87 ± 4.65% (101/119) ^b^	35.64 ± 4.70% (36/101) ^a^	88.89 ± 7.35% (32/36) ^b^
10 μM	108	91.67 ± 3.71% (99/108) ^a^	42.42 ± 4.85% (42/99) ^a^	95.24 ± 7.23% (40/42) ^a^
15 μM	134	64.18 ± 4.32% (86/134) ^c^	27.91 ± 3.81% (24/86) ^c^	83.33 ± 7.06% (20/24) ^c^

^a, b, c^ Values with different superscripts represent significant differences between groups (*p* < 0.05).

**Table 2:** Effect of linoleic acid on the developmental ability and survival rate of bovine embryos.

Groups	No. COCs	Cleavage Rate	Blastocyst Rate	Survival Rate
0 μM	165	80.00 ± 6.59% (132/165) ^b^	33.33 ± 2.41% (44/132) ^b^	86.36 ± 7.15% (38/44) ^b^
50 μM	188	61.17 ± 5.85% (115/188) ^c^	27.83 ± 1.68% (32/115) ^c^	81.25 ± 7.46% (26/32) ^c^
100 μM	151	88.74 ± 6.73% (134/151) ^a^	38.81 ± 2.55% (52/134) ^a^	96.15 ± 8.53% (50/52) ^a^
500 μM	147	60.54 ± 5.68% (89/147) ^c^	29.21 ± 1.84% (26/89) ^c^	80.77 ± 7.21% (21/26) ^c^

^a, b, c^ Values with different superscripts represent significant differences between groups (*p* < 0.05).

**Table 3:** Effect of forskolin and linoleic acid on the developmental ability and survival rate of bovine embryos.

Groups	No. COCs	Cleavage Rate	Blastocyst Rate	Survival Rate
Control	262	75.57 ± 7.46% (198/262) ^c^	30.30 ± 2.65% (60/198) ^c^	80.65 ± 7.68% (25/31) ^b^
Forskolin (10 μM)	284	82.39 ± 8.52% (234/284) ^b^	39.74 ± 4.12% (93/234) ^b^	90.24 ± 7.31% (37/41) ^b^
Linoleic acid (100 μM)	260	83.08 ± 8.46% (216/260) ^b^	37.97 ± 4.03% (82/216) ^b^	89.47 ± 8.15% (34/38) ^b^
F-L	306	89.54 ± 7.62% (274/306) ^a^	45.26 ± 5.24% (124/274) ^a^	98.18 ± 8.64% (54/55) ^a^

^a, b, c^ Values with different superscripts represent significant differences between groups (*p* < 0.05).

**Table 4:** Data analysis of lipid molecules with a significant difference between the F-L group and the control group.

Number	the Adduct Ion Form of Lipid	Cal *m*/*z*	(t/min)	VIP	FC	*p*-Value
1	PA (18:1e_10:4) − 2H	283.169	4.75	1.64	0.47	<0.01
2	DG (4:0_10:4) + H	309.170	1.18	1.70	9.25	<0.01
3	DG (6:0_11:3) + Na	375.214	1.17	6.92	11.21	<0.01
4	DG (6:0_14:2) + NH4	414.321	1.88	2.82	2.42	0.03
5	DG (22:2) + NH4	442.353	1.88	1.80	2.47	0.02
6	DG (27:4) + H	491.373	1.46	3.21	0.01	<0.01
7	DG (42:7e) + NH4	698.608	13.38	1.73	0.36	<0.01
8	LPC (16:0) + H	496.340	3.56	9.43	0.01	<0.01
9	LPC (18:0e) + H	510.392	3.64	1.02	2.15	<0.01
10	LPC (20:3) + H	546.355	3.03	1.08	0.77	0.04
11	Car (d45:3) + Na	710.642	14.89	2.50	0.48	<0.01
12	MG (18:3) + NH4	370.295	1.93	1.18	2.10	0.04
13	PC (8:0e_8:0) + H	496.340	3.59	11.20	0.01	<0.01
14	PC (8:0e_10:1) + H	522.355	2.20	2.50	0.83	0.02
15	PC (10:0e_10:3) + H	546.355	3.62	1.35	12.56	<0.01
16	PC (12:1e_11:4) + Na	606.353	1.89	1.10	2.74	0.03
17	PE (16:0e_18:3) + Na	722.510	1.61	1.87	2.31	0.04
18	SM (d18:0_16:1) + H	703.575	9.68	6.67	0.72	<0.01
19	SM (d16:0_20:4) + H	725.559	9.12	1.51	0.80	0.02
20	TG (4:0_8:0_10:1) + NH4	458.348	1.84	3.90	2.44	0.03
21	TG (4:0_8:0_11:2) + NH4	470.348	1.23	1.41	0.47	0.01
22	TG (6:0_12:0_12:3) + H	549.415	1.61	2.34	0.08	<0.01
23	TG (6:0_11:1_18:1) + NH4	638.535	6.47	1.72	188.65	<0.01
24	TG (6:0_11:3_18:2) + H	615.462	6.44	2.83	237.56	<0.01
25	TG (48:11) + NH4	802.598	7.37	1.03	0.38	<0.01
26	WE (38:2) + H	561.561	16.33	2.24	0.86	0.01

Note: FC indicates fold change, VIP indicates the variable importance for the projection.

**Table 5:** Statistics of different expressions of mRNA of the FL and Con groups.

Gene		Sample					Up/Down
	FL 1	FL 2	FL 3	Con 1	Con 2	Con 3	
*LRPAP1*	2467	1571	1888	1006	996	1040	up
*APRT*	580	462	498	117	420	127	up
*NFKBIA*	603	988	835	315	420	453	up
*CRISP2*	250	281	328	127	59	127	up
*NDRG4*	187	74	256	67	62	29	up
*TGM1*	227	1319	456	250	342	189	up
*ACSL1*	1688	945	913	664	308	540	up
*CASP7*	479	285	257	171	132	146	up
*SEC22B*	1494	1150	984	558	716	771	up
*CDC25B*	560	535	234	190	95	81	up
*MX2*	54	46	33	6	25	5	up
*EPHA4*	288	131	180	105	86	45	up
*ACADL*	289	135	140	66	99	44	up
*SLC38A5*	26	15	98	10	1	13	up
*CLDN1*	1868	1435	647	302	920	602	up
*TBC1D1*	691	581	682	381	253	285	up
*NSUN3*	560	363	339	229	241	193	up
*CREM*	42	9	89	7	13	6	up
*BMP7*	107	35	82	41	6	10	up
*IFIT5*	452	170	291	124	85	91	up
*PPARA*	37	35	31	2	0	7	up
*IRF8*	1238	1299	420	653	388	189	up
*NR2F2*	79	7	110	8	9	4	up
*SLC2A4*	141	100	29	38	37	9	up
*GPX7*	92	94	99	31	20	39	up
*ADCY8*	28	6	0	0	1	0	up
*DECR1*	256	389	215	171	142	111	up
*TGFBR2*	379	142	281	70	173	71	up
*IDH1*	9515	7924	6175	5525	3145	4063	up
*CAT*	7923	8367	5654	3618	3614	4907	up
*DCAF11*	866	680	665	394	480	354	up
*TUBA4A*	855	597	787	377	247	500	up
*ARID1A*	391	324	367	516	516	483	down
*KAT6B*	711	641	639	892	653	1606	down
*ACSF3*	56	91	22	93	133	143	down
*GLS2*	11	25	11	78	58	35	down
*CDKN2C*	2	7	9	17	25	24	down
*PLPP3*	183	139	74	280	308	363	down
*DDX54*	1533	538	850	1523	1532	1475	down
*PLN*	27	6	7	81	25	49	down
*TIAM1*	458	531	619	783	874	783	down

**Table 6:** Primers used in the present study.

Gene	NCBI Accession	Primer (3′–5′)	Size (bp)
*ACSL1*	*NM_001076085*	*TTTGTCCACGGAGAGAGCTT*	108
		*TTCAAAGGAGCCCACAATGC*	
*DECR1*	*NM_001075423*	*ACAACTTGTCTGTCCAGCCT*	136
		*GGTCCCTCACATCACACTGA*	
*ACADL*	*XM_006935526*	*TGATTCCTCACCACGCAGAA*	85
		*CCGAGAAGTCCTTGTTTGCC*	
*GPX7*	*NM_001101113*	*CGACAGCAACAAGGAGATCG* *TGATTTCCTCCACCGACACA*	224
*PPARA*	*NM_001034036*	*TTCCCTCTTTGTGGCTGCTA*	112
		*TAGGTGGAGTTTGAGCACGT*	
*TUBA4A*	*XM_045034511*	*TGGAACATGGGATTCAGCCT*	152
		*ATCAATCACAGTGGGCTCCA*	
*CAT*	*NM_001035386*	*CTGTGAACTGTCCCTACCGT*	159
		*CAGAGAAGTGGGTCCTGTGT*	
*CDKN2C*	*NM_001101054*	*CTGCAATGAATGTGGGGAGG*	229
		*TGAGACTGGCAAAGGGAGAG*	
*β-ACTIN*	*NM_173979*	*CAAGTACCCCATTGAGCACG*	159
		*GTCATCTTCTCACGGTTGGC*	

## Data Availability

The data used to support the findings of this study are included within the article.
